# TGF-beta expression during rat pregnancy and activity on decidual cell survival

**DOI:** 10.1186/1477-7827-3-20

**Published:** 2005-05-31

**Authors:** Carl Shooner, Pierre-Luc Caron, Guylaine Fréchette-Frigon, Valérie Leblanc, Marie-Claude Déry, Eric Asselin

**Affiliations:** 1 Département de Chimie-Biologie, Groupe de Recherche en Biopathologies Cellulaires et Moléculaires, Université du Québec à Trois-Rivières, C.P. 500, Trois-Rivières, Québec, G9A 5H7, Canada

## Abstract

**Background:**

During early rat pregnancy, trophoblast of the tiny embryo joins with the endometrium and epithelial cells undergo apoptosis. Near the end of pregnancy, regression of the decidua basalis (DB) is also observed (from day 14 to 20). However, little is known about the intra-cellular and molecular mechanisms involved in apoptosis regulation in the uterus during pregnancy. The objective of the present study was to investigate the presence and the developmental expression of transforming growth factor-beta isoforms (TGF-beta well known differentiation factor) in the rat endometrium throughout pregnancy and its action in vitro using cultured endometrial stromal cells.

**Methods:**

In vivo: Rats were killed at different days of pregnancy (days 2–20) and uteri removed to collect endometrial protein extracts or the uteri were fixed, embedded and sectioned for immunohistochemistry (IHC) and in situ cell death analyses using TdT-mediated dUTP nick end labeling (TUNEL). In vitro: Rats were ovariectomized and decidualization was induced using sex steroids. Endometrial stromal decidual cells were then collected and cultured.

**Results:**

An increase of apoptosis in the DB on days 14, 16 and 18 was observed. Cleaved caspase-3 was clearly detected during regression of the DB by Western analysis and immunofluorescence. Western analyses using endometrial protein extracts demonstrated that TGF-beta1, TGF-beta2 and TGF-beta3 were highly expressed at the time of DB regression (day 14). During early pregnancy, TGF-beta1 and -beta2 expressions raised at days 5.5 to 6.5. TGF-beta3 protein was not detected during early pregnancy. IHC analyses revealed that TGF-beta1 and -2 were found surrounding both epithelium (luminal and glandular) in the stroma compartment at the implantation site, and TGF-beta3 was mainly located surrounding endometrial epithelium in the stroma compartment. Smad2 phosphorylation was increased at the time of DB regression. In vitro studies using decidual endometrial stromal cells revealed that TGF-beta1 induced apoptosis and Smad2 phosphorylation. Moreover, TGF-beta1 reduced both Akt (a well known survival factor) phosphorylation and XIAP (X-linked inhibitor of apoptosis protein) expression in decidual endometrial stromal cells in vitro.

**Conclusion:**

Taken together, these results suggest that TGF-beta isoforms are regulated differently during pregnancy and may have an important role in the control of apoptosis and cell survival at specific stages during pregnancy.

## Background

Apoptosis is a type of programmed cell death and is a natural phenomenon occurring when the cells are subjected to stress such as DNA damage, death signals or lack of growth factor. Apoptotic stimuli allow an intracellular cascade of signals such as the caspases, a family of cysteine proteases implicated in the cleavage of a number of important proteins which results in cell disassembly and cell death, phagocytosis and removal of cell debris by immune cells. Apoptosis plays an important role during embryo implantation in rodents where morphological characteristics of apoptosis are observed in endometrial epithelial cells at the embryo implantation site [1-3]. Moreover, this phenomenon also occurs during late pregnancy, especially during regression of the decidua basalis (DB) in the rat endometrium [4,5]. Two decidual zones are formed during pregnancy: the primary decidual zone on the antimesometrial side of the uterus and the secondary decidual zone (or antimesometrial decidua) which is formed following expansion of the primary decidual zone [5,6]. The secondary decidual zone eventually transforms stromal cells in the mesometrial region to form the DB that regresses following day 14 of pregnancy [7]. Whether the phenomenon of growing size of embryo is a cause or correlation to the increase of apoptosis remains to be elucidated.

The first members of the transforming growth factor-beta (TGF-β) superfamily were identified on the basis of their ability to induce a transformed phenotype of certain cells in culture [8]. They are now known as multifunctional polypeptides involved in the regulation of cell proliferation and differentiation, immunoregulation, angiogenesis and the regulation of extracellular matrix [9,10]. They act via cellular signalling through Smads and Phosphorylated-Smads (P-Smads), the active form of Smads. Those proteins are translocated to the nucleus and activate transcription factors which in turn activate caspases and other regulation proteins [11,12]. Another characteristic of TGF-β is its capacity to induce apoptosis in several cell types [7,13]; in fact, TGF-β was shown to have a pro-apoptotic function mediated by caspases [14-16]. Genes encoding the three isoforms are localized on different chromosomes and the isoforms molecular weights are slightly different: 15, 12.5 and 12 KDa for TGF-β1, β2 and β3 respectively; they share 80 % sequence identity and are produced in latent forms which are activated into a 112-amino acid mature peptide [17]. Multiplicity of TGF-β isoforms and sequence conservation within each form through evolution suggests important specific roles. Moreover, it has been demonstrated that TGF-β1, -β2 and -β3 are differently expressed in the mouse uterus [18] and porcine conceptus-maternal interface [19,20].

The uterus is a hormone-dependent organ and it is subject to an abundant amount of cellular proliferation and cell death. Studies have shown that apoptosis is increased in the rat endometrium during implantation and regression of the decidual basalis in the rat [1,2,21]. The mRNA for TGF-β1 has been shown to be present within the uterus during rat pregnancy and was localized to the luminal and glandular epithelial cells during early and late pregnancy [22]. TGF-β1 and -β2 mRNAs were also found in the mouse uterus during pregnancy [23-25]. Expression of TGF-β2 and TGF-β3 mRNAs were also shown to be expressed in the mouse periimplantation uterus [18]. A study showed that TGF-β1 and -β2 treatments on cultured endometrial rat stromal cells induced apoptosis [7]. These studies suggest that TGF-β isoforms might be involved specifically in the control of apoptosis in the uterus during pregnancy. However, the mechanisms involved in the control of apoptosis in the endometrium during pregnancy are poorly documented. Studies suggesting the importance of TGF-β isoforms at specific time of pregnancy were done at the mRNA level and it is important to determine their presence at the protein level. Thus, the aim of this study was to determine the expression and the developmental expression of TGF-β1, β2 and β3 proteins in the rat uterus throughout pregnancy and to further determine *in vitro*, the effect of TGF-β in determining decidual cell fate. We found that the three isoforms of TGF-β were expressed and regulated differently in epithelial and stromal endometrial cells during rat pregnancy and showed using primary decidual cell cultures the involvement of TGF-β in the regulation of programmed cell death. Moreover, the present study shows that TGF-β signals through Smad2, which coincide with XIAP and Akt down regulation and induction of apoptosis.

## Methods

### Reagents

TGF-β1 (sc-146, lot # F262, 200 μg/ml), TGF-β2 (sc-90, lot # B202, 200 μg/ml) and TGF-β3 (sc-82, lot # A222, 200 μg/ml) polyclonal antibodies were purchased from Santa Cruz Biotechnology, Inc (Santa Cruz, CA, USA). CDC47/MCM7 antibody was obtained from Medicorp (Montréal, QC, Canada). Phospho-Akt (Ser 473), Akt, XIAP, Cleaved caspase-3, and Phospho-Smad2 (Ser 465 / 467) antibodies were obtained from Cell Signaling Technology (Beverly, MA, USA). The Keratin 8/18 antibody used to determine cell culture purity was donated by Dr Monique Cadrin (Univ. of Québec at Trois-Rivières, QC, Canada). Anti-Smad2/3 antibody was purchased from Calbiochem (San Diego, CA, USA). Vectastain ABC Kit for rabbit IgG was purchased from Vector Laboratories Inc. (Burlingame, CA, USA). *In Situ *Cell Death detection kit (TUNEL), POD and DAB substrate was obtained from Roche (Laval, QC, Canada). TGF-β1 recombinant protein was purchased from Biosource (Cat # PHG9104, lot # 16865-01S, 5 μg, diluted at 50 μg/ml, QC, Canada).

### Animals

Sprague-Dawley female rats, 200–225 g, were obtained from Charles River Laboratories Canada. Animals were maintained on standard chow and water, which were available *ad libitum*, in animal facilities illuminated between 6:00 h and 20:00 h. All procedures were performed in accordance with guidelines of the Canadian Council on Animal Care for the handling and training of laboratory animals and the Good Health and Animal Care Committee of the Université du Québec à Trois-Rivières. Male and female mice were mated overnight and confirmation of pregnancy was determined by vaginal smears and/or the presence of a vaginal plug (day 1). Rats were killed on day 2, 4, 5, 6, 8, 10, 12, 14, 16, 18 and 20 of pregnancy at 10:00 h in the morning and at 18:00 h for days 5.5 and 6.5. Six to 8 different rats were used for each time of pregnancy. Uteri were collected and fixed for immunohistochemical staining (IHC) and apoptotic cell death detection by [TdT]-mediated deoxyuridinetriphosphate nick end-labeling (TUNEL) or endometrial protein extracts collected for Western blot analysis.

### Rat pretreatments and decidual endometrial stromal cell culture

A total of 10 rats were ovariectomized and then allowed to recover from surgery for a minimum of 10 days. They were pre-treated with physiological doses of estradiol (1,3,5(10)-Estratriene-3,17β-diol, Sigma-aldrich) and progesterone (Laboratoire Mat, PQ) to induce decidualization as described previously [26]: 1) 0.2 ug estradiol injection per day for three days (in the morning, day -2,-1 and 0); 2) On the third day (day 0 of pseudopregnancy), another injection in the afternoon of estradiol (0.2 μg) and progesterone (1 mg) was performed; 3) No treatment for 2 days (day 1 and 2 of pseudopregnancy); 4) Injections of estradiol (0.1 μg) and progesterone (4 mg) for three days (day 3, 4 and 5 of pseudopregnancy); 5) Another injection of estradiol (0.1 μg) in the afternoon on day 7 (day 4 of pseudopregnancy); 6) Rats were killed on day 8 (day 5 of pseudopregnancy). All endometrial stromal cells collected for cultures were recovered from rats treated with the protocol described above.

Uteri were removed and horns taken and immerged in HBSS solution containing HEPES (20 mM), penicillin (100 units/ml), streptomycin (100 μg/ml) and fungizone (1,25 μl/ml) (Invitrogen, ON, Canada). Further manipulations were performed in a sterile environment. The uterine horns were transferred into a sterile petri containing HBSS, slit longitudinally and immersed in trypsin type I solution (0.3%) (Roche Diagnostics, QC, Canada) in HBSS and agitated for 60 minutes at room temperature. Uterine horns were then vortexed at maximum for 5 sec and supernatant containing epithelial cells was discarded. Uterine horns were washed three times with 2.5 ml of HBSS and immersed in a HBSS solution containing trypsin type I (0.03%), DNAse I (0.016%) and collagenase type II (0.064%) for 15 minutes at 37°C in a water bath. Uterine horns were then vortexed at maximum for 5 sec. The supernatant containing stromal cells was transferred into a sterile falcon tube containing 150 μl of FBS D.C (Dextran-Charcoal extracted). Uterine horns were washed two times with 2.5 ml of HBSS and the supernatant was mixed with stromal cells. Uterine horns were discarded and stromal cells were centrifuged at 1000 g for 5 minutes. Cells were washed two times with HBSS and centrifuged. The supernatant was discarded and cells diluted with DMEM-F12 (Ph 7.1) (Invitrogen, ON, Canada) containing 2.438 g/L NaHCO_3_, 10% FBS D.C. and gentamycine 50 μg/ml. Cells were incubated at 37°C in an atmosphere of 5% CO_2_. Cells were plated in 6-well plates (Corning plates) at a density of 50% (4 × 10^5 ^cells per well). The medium was changed two hours after the first incubation in order to eliminate epithelial cell contamination from stromal cell cultures. The purity of stromal cells was more than 97%: cell culture contamination with epithelial cells was evaluated by cellular morphology and immunofluorescence using a Keratin 8/18 antibody. Three to 5 days after plating (more than 90% of confluency reached), cells were treated for 24 hours in the presence or absence of increasing doses of TGF-β recombinant protein. Total proteins from treated cell cultures were extracted using TRIZOL (Invitrogen, ON, Canada). For Western blot analyses, 15 μg of total protein was used for each analysis.

### Immunohistochemical staining

The uterus was fixed in 4% paraformaldehyde solution and embedded in paraffin. Tissue sections 7 μm thick were mounted on polylysine-coated slides, deparaffinized, rehydrated, and then heated in 10 mM citrate buffer (pH 6) containing triton X-100 (Sigma-Aldrich) 0.1% (v/v). After two washes with PBS, slides were then incubated with 0.3 % hydrogen peroxide in methanol for 30 min to quench endogenous peroxidase activity. After washing with PBS, tissues were incubated with blocking serum (Vectastain ABC Kit) at room temperature for 1 h. Then, a primary antibody diluted in blocking serum (TGF-β1, β2 or β3; 1:50 dilution or CDC47/MCM7; 1:100 dilution) was added to the slides and incubated at 4°C overnight in a humidified chamber. After washing 5 min. in PBS, tissue sections were incubated for 30 min. with 3 μg/ml biotinylated antibody (anti-rabbit or anti-mouse). Subsequently, slides were washed with PBS and incubated with avidin-biotin complex reagent containing horseradish peroxidase for 30 min. Slides were washed with PBS for 5 min and color development was achieved using DAB substrate. The tissue sections were counterstained with haematoxylin. Negative controls were performed using the same protocol but substituting the primary antibody with normal rabbit IgG (Vector Laboratories Inc., Burlingame, CA, USA).

### Immunofluorescence

Tissues were prepared as described in the immunohistochemical section. Cleaved caspase-3 antibody was diluted 1:100 in blocking serum and slides were incubated at 4°C overnight. After washing twice for 5 min. in PBS, tissue sections were incubated for 30 min. at room temperature with 2 mg/ml Alexa Fluor 488 donkey anti-rabbit (1:50). Subsequently, slides were washed with PBS and mounted. Negative controls were performed using the same protocol but substituting the primary antibody with normal rabbit IgG. Sections were examined using an OlympusBX60 microscope equipped with a Coolsnap-pro CF digital camera (Carsen Group, ON, Canada).

### TdT-mediated deoxyuridinetriphosphate nick end-labeling (TUNEL)

Tissue sections were deparaffinized, rehydrated and rinsed with PBS. They were incubated with proteinase K (20 μg/ml) for 30 min. at room temperature. Slides were washed twice with PBS, the endogenous peroxidase was quenched with 0.3 % hydrogen peroxide in methanol for 30 min. The slides were rinsed and incubated with 10 mM citrate solution for two min on ice. Then, tissue sections were rinsed with PBS and incubated with TdT labelling reaction (*In Situ *Cell Death Detection, POD) for 30 min at 37°C in humidified environment. Slides were washed three times in PBS and tissue sections were blocked with 3% BSA for 20 min. at room temperature. Converter-POD solution was added to the slides and incubated for 30 min. at 37°C in humidified environment. Slides were washed for 5 min. in PBS, colour development was achieved using DAB substrate and counterstained with haematoxylin. Negative controls were performed using the same protocol without TdT enzyme.

### Protein extraction and Western analysis

Protein homogenates from pregnant endometrium were isolated according to a protocol previously described [27]. Briefly, uteri from Day 2 to Day 20 pregnant rats were rapidly excisedand placed in ice-cold saline until dissected. Uteri were carefully laid on a glass plate and placed on the stage of a dissecting microscope. In early pregnancy (Day 2 to 5.5), total endometrium was scraped using a microscope glass and collected. Uteri from Day 6 to 10 the placenta and decidua were at an early stage of differentiation and could not be reliably separated. For this reason, DB dissectedfrom animals between these days of pregnancy contain some chorioallantoic cells, but antimesometrial decidua, choriovitelline tissues, fetus, and myometrium were removed. Even though we carefully dissected DB from these tissues, it is a possibility that a contamination with some antimesometrial decidual cells that regress to form the deciduas caspularis (DC) would occur. This is an important fact that we need to take into consideration. In uteri collected from Day 12 to 20 pregnant rats, DB were isolated by gently separating the placenta and myometrial regions with 23-gauge needles. Additionally, the DB began to regress on Day 14 and became too thin to reliablydissect after Day 17. The protocol for DB isolation was described previously by Ogle and George [28].

Endometrial cells from pregnant animals were homogenized using a pipette in RIPA lysis buffer (PBS 1× pH 7.4; 1% Nonidet P-40; 0.5% Sodium deoxycholate; 0.1% SDS; Protease Inhibitor Cocktail Tablets (Roche Diagnostics Canada, PQ)). Homogenates were centrifuged (12,000 × g for 20 min at 4°C) to remove insoluble material. The supernatant was recovered and stored at -20°C pending analysis. Protein content was determined with the Bio-Rad DC Protein Assay. Protein extracts (50 μg) were heated at 94°C for 3 min, resolved by 10% SDS-PAGE and electrotransferred to nitrocellulose membranes using a semidry transfer (Bio-Rad, Mississauga, ON). The membranes were then blocked 2 h at room temperature with PBS containing 5 % milk powder, then incubated with anti TGF-β 1-2-3 1:1000 ; P-Smad2 (Ser 465 / 467) 1:1000 and Smad 2/3 1:1000 and subsequently with horseradish peroxidase-conjugated anti-rabbit or anti-mouse secondary antibody (1:3000; room temperature for 45 min). All membranes were stripped with Restore western blot stripping buffer (Pierce, # 21059, lot # FH71541), reprobed with an antibody specific to β-actin which was used as an internal standard. Peroxidase activity was visualized with the Super signal^® ^West Femto maximum sensitivity substrate (Pierce, Arlington Heights, IL, USA), according to the manufacturer's instructions. Signal was visualized using the Biochemi Imaging System (UVP, CA, USA). Densitometrical analyses were performed (protein of interest and β-actin) using the GelDoc 2000 and the Quantity One software (Bio-Rad, Mississauga, ON, Canada). Results are expressed as a ratio (protein of interest/β-actin) to correct for loading for each endometrial sample.

### Hoechst and trypan blue exclusion staining

Following TGF-β treatment, both floating and attached cells were resuspended in PBS containing Hoechst 33258 for 24 hours at 4°C or resuspended in trypan blue solution (0,4%) for 5 minutes. Hoechst nuclear staining was viewed and photographed using a Olympus BX60 fluorescence microscope and a Coolsnap-Pro CF digital Camera (Carsen Group, ON, Canada). Cells with typical apoptotic nuclear morphology (nuclear shrinkage, condensation and fragmentation) were identified and counted using randomly selected fields on numbered photographic slides, of which the "counter" was not aware of the treatment, so as to avoid experimental bias. A minimum of 200 cells per treatment group were counted in each experiment. For trypan blue exclusion test, blue cells were counted under a regular microscope and were counted as non-living cells.

### Statistical analysis

Western analyses of pregnant animals were repeated six to eight times (6 to 8 different endometrial extract per day of pregnancy from 6 to 8 different rats). Endometrial extracts from each rat were assessed individually. Western analyses of cultured decidual cells were repeated 5 times for each TGF-β dose (for each culture experiment, decidual cells were recovered from a pool of ten different ovariectomized/treated rats). Results subjected to statistical analyses were expressed as mean ± SEM. Data were subjected to one-way ANOVA (PRISM software version 4.0; GraphPad, San Diego, CA). Differences between experimental groups were determined by the Tukey's test.

## Results

### Apoptosis expression during pregnancy

**Figure 1:**
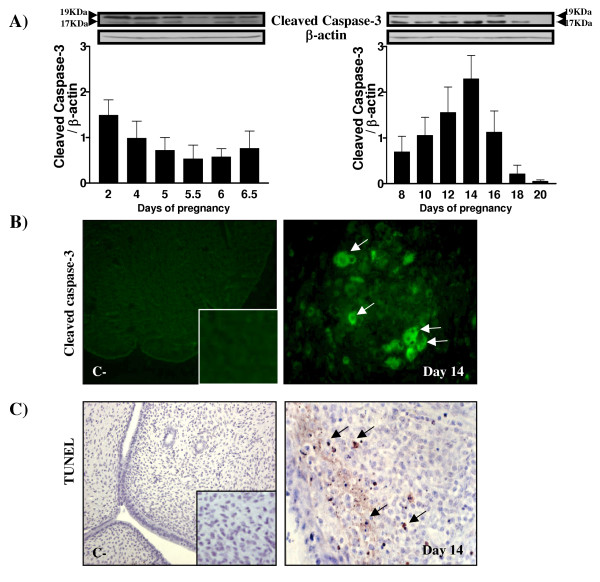
Detection of apoptosis in pregnant endometrial tissues as demonstrated by Western and TUNEL analyses. A) Apoptosis as determined by Western analysis of cleaved caspase-3 (one blot presented out of 6). β-actin blots shown were used as controls to correct for loading in each lane. Blots shown are from one representative experiment. Graphics represent Western blot densitometrical analysis of the 17 KDa cleaved fragment. B) Cleaved caspase-3 immunofluorescence at day 14 of pregnancy. Magnification: control: 10× ; inset and Day 14: 40×. C) TUNEL analysis at day 14 of pregnancy. B) and C) One representative section is presented out of 6 experiments. C-: negative control. Magnification: control: 10× ; inset and Day 14: 40×. Arrows indicate positive staining.

In order to confirm the presence of apoptosis in DB, the presence of the activated form of caspase-3 was measured by Western analysis (Fig. 1A) and immunofluorescence (Fig. 1B) using a day 14 pregnant uterine section. TUNEL measurement was also performed using a day 14 pregnant uterine section (Fig. 1C). As demonstrated by Western analysis, Fig. 1A clearly demonstrates that apoptosis was present in the endometrium at day 14: the 17 KDa cleaved caspase-3 fragment was significantly and gradually increased from day 8 and was maximal at day 14. Cleaved caspase-3 fragment was observed in the cytoplasm of apoptotic cells as demonstrated by immunofluorescence (Fig. 1B) and TUNEL positive cells were also found in the in the endometrium at day 14 (Fig. 1C).

### Expression of TGF-β1, β2 and β3 in the rat uterus during pregnancy

**Figure 2:**
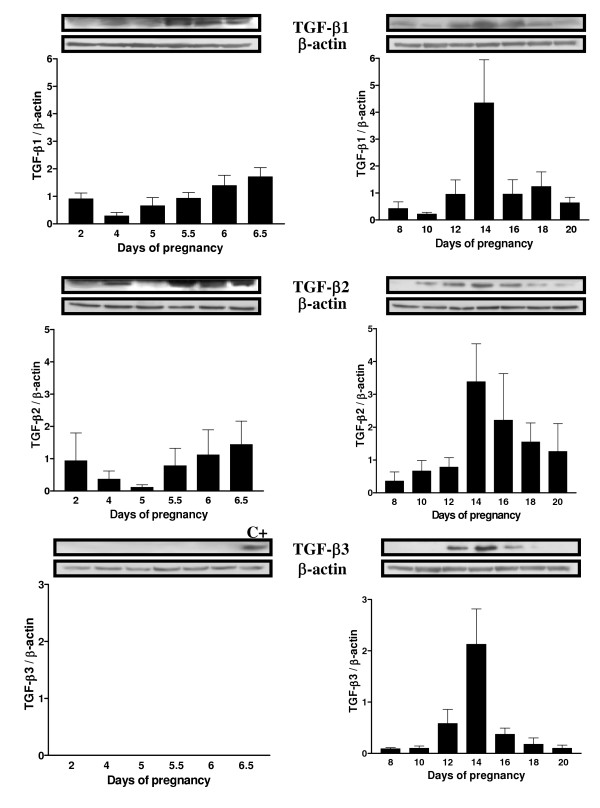
TGF-β1, -β2 and -β3 expressions in rat endometrium during rat pregnancy. Total endometrial proteins were collected at different days of pregnancy. β-actin blots shown were used as controls to correct for loading in each lane. Blots shown are from one representative experiment. Graphics represent Western blot densitometrical analysis. Data represent the mean ± SEM of six independent experiments (six different rats). C+: positive control (day 14 endometrial protein extract).

**Figure 3:**
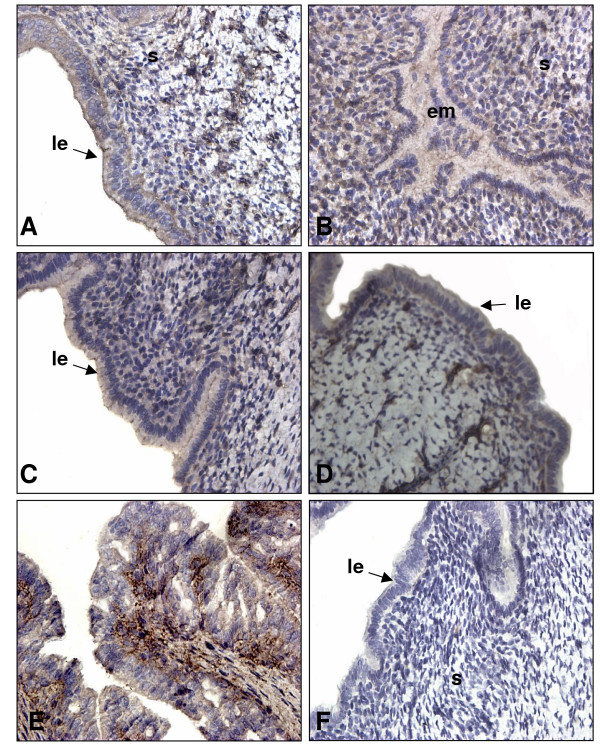
Immunohistochemistry of TGF-β1 in rat endometrium during pregnancy. IHC shown are from one representative experiment and were repeated 6 times using 6 different uterine sections from 6 different rats per day of pregnancy. Representative days of pregnancy are presented (A: day 4; B: day 5.5; C: day 6.5; D: day 10; E: day 14; F: negative control in which primary antibody was absent). le: luminal epithelium; em: embryo; s: stroma. Magnification: 40×.

**Figure 4:**
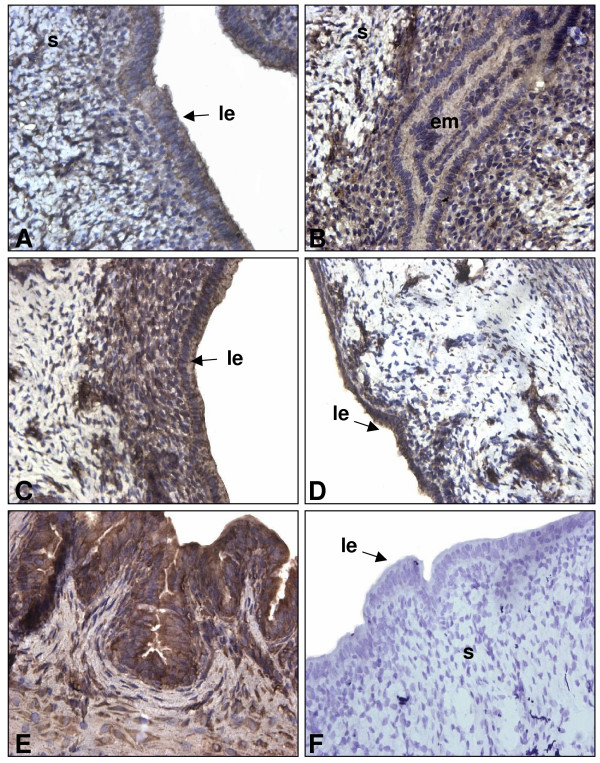
Immunohistochemistry of TGF-β2 in rat endometrium during pregnancy. IHC shown are from one representative experiment and were repeated 6 times using 6 different uterine sections from 6 different rats per day of pregnancy. Representative days of pregnancy are presented (A: day 4; B: day 5.5; C: day 6.5; D: day 10; E: day 14; F: negative control in which primary antibody was absent). le: luminal epithelium; em: embryo; s: stroma. Magnification: 40×.

**Figure 5:**
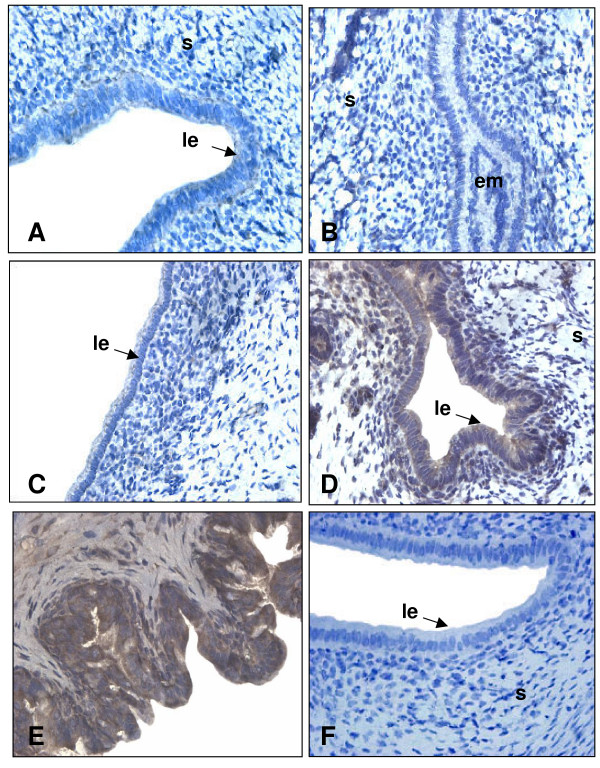
Immunohistochemistry of TGF-β3 in rat endometrium during pregnancy. IHC shown are from one representative experiment and were repeated 6 times using 6 different uterine sections from 6 different rats per of day pregnancy. Representative days of pregnancy are presented (A: day 4; B: day 5.5; C: day 6.5; D: day 10; E: day 14; F: negative control in which primary antibody was absent). le: luminal epithelium; em: embryo; s: stroma. Magnification: 40×.

To document the presence and expression of TGF-β proteins in the uterus throughout pregnancy, IHC and Western analyses were performed on uterine sections and lysate of pregnant rats respectively. It was important to document the presence of TGF-β proteins since the information found in the literature shows principally mRNA expression of different TGF-β isoforms in specific periods of pregnancy rather than in the whole gestation period. Western blot analyses shown in figure 2 demonstrate that TGF-β1 and β2 are both expressed in a similar pattern. Their expression is increased following implantation (days 5.5 to 6.5) and is maximal during regression of the DB (day 14; p < 0.01). However, the localization of the expression of those two isoforms during pregnancy is slightly different (Fig. 3 and 4 respectively): immunohistochemicals analysis confirms that during early pregnancy the signal is found in both epithelial and stromal cells, but during late pregnancy TGF-β1 is expressed mainly in stromal cells while TGF-β2 is located in epithelial cells. It is interesting to see that both TGF-β1 and β2 isoforms are clearly present in the epithelial cells and in stroma at the time of implantation (day 5.5) all around the uterine lumen surrounding the implanting conceptus. On the opposite, TGF-β3 has not been found during early pregnancy (Fig. 2 and 5). However, TGF-β3 was increased and present at the time of DB regression (days 12 to 16; p < 0.001) suggesting that its action might be limited to decidual regression during pregnancy.

### Expression of Smad2 and Phospho-Smad2 during pregnancy

**Figure 6:**
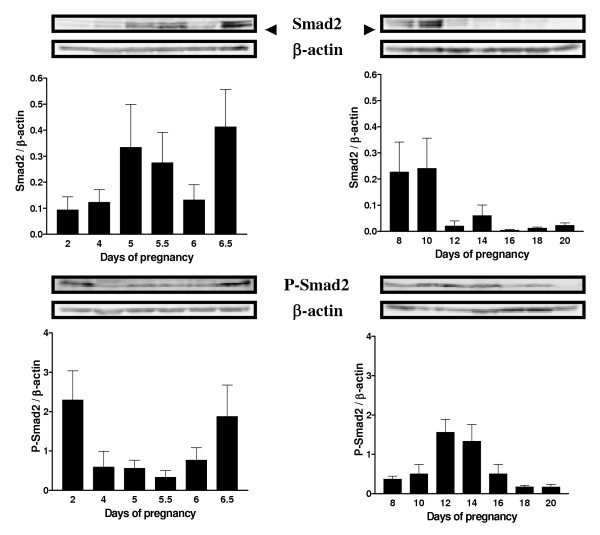
Smad2 and phospho-Smad2 (P-Smad2) expressions in rat endometrium during pregnancy. Total endometrial proteins were collected at different days of pregnancy. β-actin blots shown were used as controls to correct for loading in each lane. Blots shown are from one representative experiment. Graphics represent Western blot densitometrical analysis. Data represent the mean ± SEM of six independent experiments (six different rats).

TGF-β signal transduction is mediated intracellularly by Smad proteins, including the R-Smads (receptor regulated Smads including Smad2), the I-Smads (inhibitor Smads), and the Co-Smad (common Smad). The triggering event in Smad activation is the type I receptor-dependent sequential phosphorylation of the two C-terminal serine residues in Smads [29]. Therefore, phosphorylation of the C-terminus of receptor-activated Smads, particularly Smad2, is crucial for initiation of the TGF-β signaling. Western blot analyses of Smad2 and phospho-Smad2 on endometrial cell lysate from pregnant rats were carried out to confirm that TGF-β isoforms present in the endometrium during pregnancy have an activity on those cells. The results demonstrate that Smad2 expression is regulated throughout pregnancy (Figure 6). Its expression is high from day 5 to 10 and is dramatically reduced from day 12 to the end of pregnancy (p < 0.05). However the levels of phospho-Smad2, the activated form, are high on days 12 and 14 and are gradually reduced to the end of pregnancy. The presence of high levels of phospho-Smad2 correlates with the high expression of the three TGF-β isoforms and the presence of apoptosis, with an exception on day 2 of pregnancy (Figure 6). Although high levels of phospho-Smad2 are observed, levels of TGF-β1 and -2 isoforms are higher when compared to days 4 and 5 and caspase-3 cleaved fragment is also present.

### TGF-β action *in vitro *on decidual endometrial stromal cell fate

**Figure 7:**
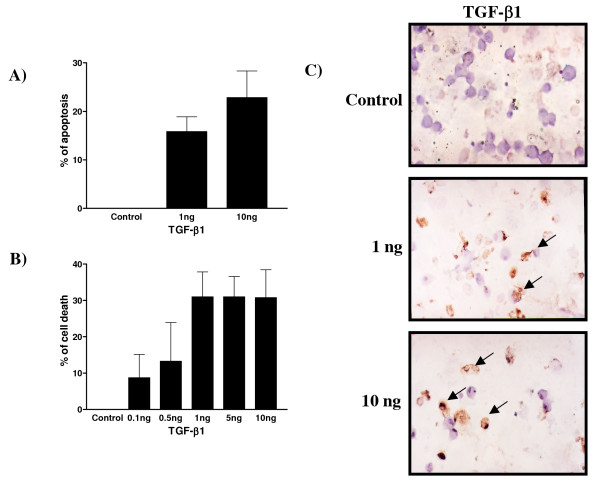
Effect of TGF-β1 (ng/ml) on cell survival in cultured rat endometrial cells as demonstrated by Hoechst staining, TUNEL and trypan blue exclusion analyses. A) Apoptosis as determined by Hoechst nuclear staining. Data represent the mean ± SEM of six independent experiments. All doses are significantly different from control (p < 0.001). B) Cell viability as determined by trypan blue exclusion assay. Data represent the mean ± SEM of six independent experiments. 1, 5 and 10 ng/ml TGF-β1 doses are significantly different from control (p < 0.0001). C) Apoptosis as determined by TUNEL assay. Representative fields are presented out of 6 experiments. Arrows indicate positive staining.

As demonstrated previously by Moulton in 1994 [7], TGF-β1 induces DNA cleavage in rat decidual cells. To confirm that TGF-β1 is responsible for the induction of apoptosis, three different techniques were used to measure apoptosis (Figure 7). Hoechst nuclear staining (Figure 7A) and TUNEL analysis (Figure 7C) clearly demonstrated that TGF-β1 induced apoptosis in a dose-dependent manner (p < 0.0001). Apoptosis was increased to 20% at 1 ng/ml of TGF-β1 and up to 30% at 10 ng/ml. Trypan Blue exclusion staining assay was used to test for viability and cell death; even though this test is not an apoptotic specific test, it shows a direct effect of TGF-β1 on cell survival and viability. A dose-dependent increase of cell death was observed in response to TGF-β1 treatment (Figure 7B).

### Effect of TFG-β on Akt, P-Akt, CDC-47 and XIAP expression in vitro on decidual endometrial stromal cells

**Figure 8:**
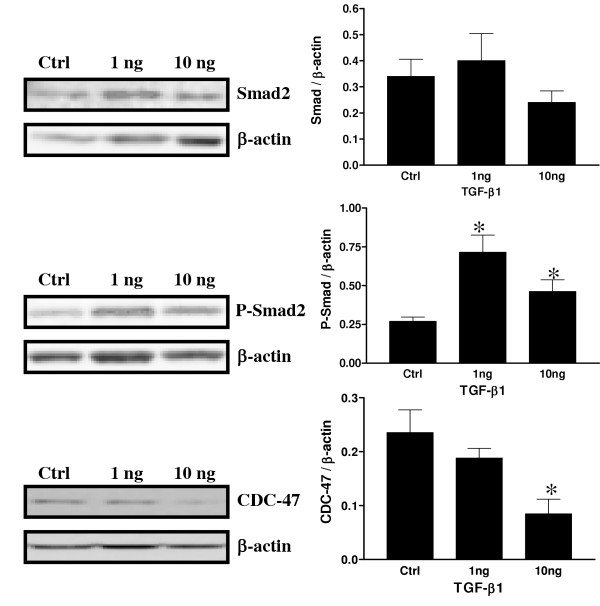
Expression of Smad2, P-Smad2 and CDC47 in cultured rat endometrial cells *in vitro *as demonstrated by Western blot analyses in response to TGF-β1 (ng/ml). β-actin blots shown were used as controls to correct for loading in each lane. Blots shown are from one representative experiment. Graphics represent Western blot densitometrical analysis and are the mean ± SEM of four independent experiments. *Significantly different from control (p < 0.05).

**Figure 9:**
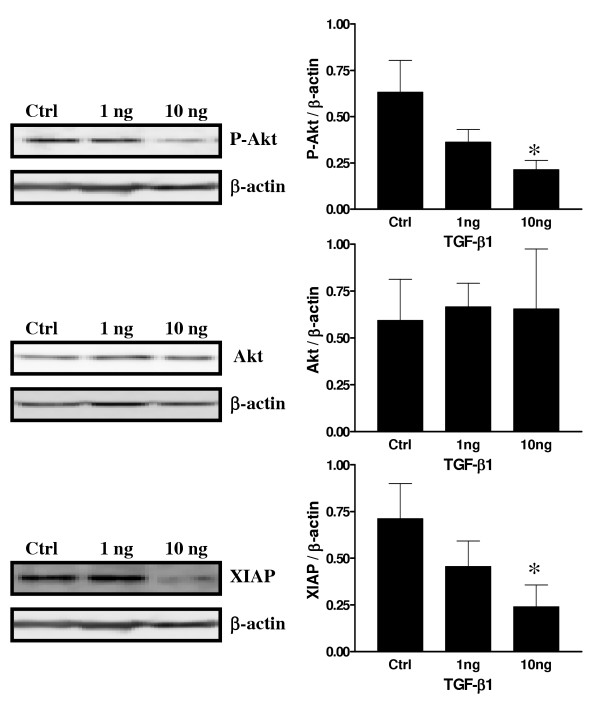
Expression of Akt, P-Akt and XIAP in cultured rat endometrial cells *in vitro *as demonstrated by Western blot analyses in response to TGF-β1(νγ/μλ). β-actin blots shown were used as controls to correct for loading in each lane. Blots shown are from one representative experiment. Graphics represent Western blot densitometrical analysis and are the mean ± SEM of four independent experiments. *Significantly different from control (p < 0.05).

To further determine how TGF-β might act at the intra-cellular level to induce apoptosis in decidual cells, experiments were carried out to determine the possible interaction of TGF-β and the PI3K/Akt survival pathway. Recent studies have suggested and demonstrated that TGF-β directly acts with Smad3 to regulate the sensitivity to TGF-β induced apoptosis [30,31] and another study showed that TGF-β exerts a largely inhibitory effect on basal meningioma proliferation possibly through Smad 2/3 [32]. Since Smad3 is directly involved in TGF-β signaling, a similar mechanism might be involved in the control of decidual cell fate. Another study has shown that Akt activity might in turn be affected by the presence or absence of inhibitor of apoptosis proteins such as XIAP [33,34]. Figure 8 shows that Phospho-Smad2, the activated form, is significantly increased in response to TGF-β1. However, the concentration-response relationship in term of Smad phosphorylation appears to be biphasic, with 1 ng/ml stimulating, while 10 ng/ml having a significant effect compared to control (p < 0.05) but lower than the 1 ng/ml dose. There was no significant difference observed in term of Smad2 protein expression in response to TGF-β1. As demonstrated in Figure 8, the proliferation marker CDC-47 was significantly decreased in response to TGF-β1 treatment. Figure 9 demonstrates that Phospho-Akt, the active form of Akt, was highly expressed in control cells, indicating that this pathway is active and important in cell proliferation and cell survival. However, in the presence of TGF-β1, Phospho-Akt was significantly decreased suggesting an interaction of TGF-β and the PI3K/Akt survival pathway. Total Akt protein expression was not influenced by TGF-β1 treatment. Moreover, XIAP protein expression, a well known inhibitor of apoptosis protein, was significantly reduced in response to increasing doses of TGF-β.

## Discussion

Transforming growth factor-β isoforms have been known to be expressed and regulated differently in several types of tissues such as ovine uterus [35], human colon carcinoma [36] and at the porcine conceptus-maternal interface [19]. They are also recognized as pro-apoptotic factors in many cell types including fetal rat hepatocytes [14] and human leiomyoma smooth muscle cells [37]. However, little is known about the role of TGF-β isoforms in the rat uterus during pregnancy. The aim of the present study was to investigate the pro-apoptotic functions of TGF-β isoforms in rat uterus during pregnancy and also to determine more precisely the different patterns of expression of those isoforms in pregnant endometrium. The results presented in this study demonstrate that, as observed in other tissues, TGF-β isoforms (β1, β2 and β3) are differently regulated in rat uterus throughout pregnancy.

It is already known that apoptosis is induced during embryo implantation and decidualization in rodents [1,2,7]. Our results confirm that apoptosis is induced in the pregnant rat uterus especially during regression of the decidua basalis. Caspases are well known executioners of apoptosis [15,38]. The highest concentration of cleaved caspase-3 protein, the activated form of caspase-3, was found on day 14 of pregnancy, at the time of DB regression. The presence of active caspase-3 shows that this pathway might be important to induce cleavage of critical survival proteins and to further stimulate apoptosis during the regression of DB. However, caspase-3 activation was weakly observed at the time of implantation and this might be explained by the fact that, although uterine epithelium undergoes degeneration in the presence of embryo [4-6], only some epithelial cells undergo apoptosis during this process at the implantation site. Western blot and IHC analyses might not be sensitive enough techniques to detect the presence of small levels of cleaved caspase-3 in epithelial cells at implantation. It is also a possibility that degeneration of the epithelium may also involve non-apoptotic pathways.

The logical pursuit of this study was to determine the expression of TGF-β isoforms during those critical stages of pregnancy. The present results are consistent with previous work published in regards to TGF-β isoforms in other systems and in mouse uterus [18,22,39], where expression and regulation is different for each isoform. TGF-β1 was already known to induce apoptosis in human [40] and rat [7] endometrial stromal cells. In the present study, TGF-β1 and TGF-β2 were found throughout pregnancy and were particularly strong during apoptotic phases such as regression of the DB. On the other hand, using both IHC and Western analyses, TGF-β3 protein was undetectable in the early stages of gestation, suggesting that TGF-β3 may not be required in the regulation of cell death during embryo implantation and early pregnancy. This result supports a study performed by Das et al. [18] showing that TGF-β3 mRNA was absent during early pregnancy. Nevertheless, the three mammalian forms of TGF-β are strongly expressed during regression of the DB suggesting they could all play an important role during the regulation of programmed cell death to induced regression of DB. Those three isoforms are also expressed differently in distinct cells of the same tissues; similarities have been observed in human normal and malignant prostate epithelial cells [39] as TGF-β2 and TGF-β3 are more expressed in epithelial cells than in stromal cells during late pregnancy. The strong expression of TGF-β1 during rat early pregnancy (day 5.5 to 6.5) may be explained by the fact that this isoform could be necessary to initiate embryo implantation processes during the periimplantation period, a similar situation that is observed during trophoblast invasion at the time of human embryo implantation [41].

The next logical step of the present study was to determine if the TGF-β isoforms present during pregnancy were active. A lot of information can be found in many physiological systems regarding TGF-β and its cellular receptors [8,10] and Smad proteins responsible for its intracellular signal transduction [42-44]. Recent studies have suggested and demonstrated that TGF-β directly acts with Smad3 to regulate the sensitivity to TGF-β induced apoptosis [30,31] and another study showed that TGF-β exerts a largely inhibitory effect on basal meningioma proliferation possibly through Smad 2/3 [32]. Since Smad3 is directly involved in TGF-β signaling, a similar mechanism might be involved in the control of decidual cell fate. Since it is well known that TGF-β signals through Smads proteins, Western blot and IHC analyses were performed on pregnant endometrial cell lysates to measure Smad2 and phospho-Smad2 (the activated form) to test this hypothesis. The results showed that Smad2 protein was increased and stronger at the time of embryo implantation and that Smad2 phosphorylation was gradually increased during regression of the DB, which correlates with the presence of TGF-β isoforms. These results suggest that TGF-β isoforms present during these two critical stages of pregnancy might act through Smads proteins to induce apoptosis or to induce other genes known to be regulated by TGF-βs. Smad2 protein is relatively low during regression of the DB compared to early stages of pregnancy. This result is supported by a recent study showing that increased Smad2 expression is probably caused by the invasion of the trophoblast resulting in the formation of the first decidual zone [45]. However, Smad2 phosphorylation is not as much increased during early pregnancy as compared to late pregnancy. It is again a possibility that, since only epithelial cells undergo apoptosis at the time of embryo implantation, the possible increase of Smad2 phosphorylation induced by TGF-β might only be observed in a small number of cells which was undetectable with the techniques used.

To better understand the effect of TGF-β at the cellular level, decidual cell cultures were used to further investigate the interaction of TGF-β and the PI 3-K/Akt survival pathway. Although a recent study showed the possibility that cells obtained following artificial decidualization might be different from those obtained from pregnant animals [46], this model is an excellent alternative to obtain sufficient material to test the role of TGF-β and to correlates the data with the physiologic situation. It has been demonstrated recently that Akt phosphorylation is directly induced by 17β-estradiol in the ovariectomized rat uterus indicating that sex steroids have an important influence on endometrial cell fate [47]. Very recent studies have shown that TGF-β might directly block Akt activity through Smad activation [30]. Thus, stromal decidual cells were treated with different doses of TGF-β1 to determine if Smad activation might in turn block Akt survival pathway to induce cell death. TGF-β1 induced Smad2 phosphorylation in cultured endometrial stromal cell *in vitro *and triggered apoptosis in a dose-dependent manner, which was accompanied by a reduction of cell proliferation, confirming TGF-β as an apoptotic factor in rat decidual endometrial stromal cells. In response to TGF-β1, Akt phosphorylation was significantly decreased indicating that Akt activity inhibition might be an important mechanism for TGF-β-induced apoptosis in this model. Other studies have shown that Akt activity might in turn be affected by the presence or absence of inhibitor of apoptosis proteins such as XIAP [33,34] and cIAP-1 [48]. The results support the hypothesis that TGF-β action via Smads not only acts at the transcriptional level to induce production of apoptotic factors in decidual cells but also at the protein level to block activation of survival factors.

Akt phosphorylation was recently shown to be regulated by XIAP, a well known inhibitor of apoptosis protein, in human ovarian epithelial surface cells and in rat granulosa cells [33,34]. The present results show a possible interaction (direct or indirect) between TGF-β1 and XIAP protein expression. TGF-β1 reduced XIAP expression *in vitro *in a dose dependent manner. Recent studies have demonstrated that XIAP can act as a cofactor in the regulation of gene expression induced by TGF-β and is independent of Smad4 [14,49]. Because TGF-β1 reduced Akt phosphorylation and that XIAP was shown to induce Akt phosphorylation [34], it is possible that TGF-β action in this case might be through Smad2 activation and XIAP gene expression which in turn would act on Akt phosphorylation. Further experiments will be necessary to have a better understanding of the interactions between TGF-β and Akt survival pathway. In particular, how specifically Smads act on XIAP expression and how XIAP acts on the PI 3-K/Akt survival pathway activity. Other factors such as Smac/Diablo, a XIAP intracellular inhibitor, which was demonstrated as being regulated by 17β-estradiol in the rat during estrous cycle [50], might also be a putative candidate for regulation of TGF-β activity in decidual cells.

## Conclusion

In conclusion, this study demonstrates that the three isoforms of TGF-β are differently localized and regulated in endometrial cells of pregnant rats, particularly at the time of implantation and regression of the DB. The present study also showed that TGF-β plays an important role in the control of cell survival and cell death and that it may interacts with the PI 3-K/Akt survival pathway through Smad activation to allow apoptosis induction. Further experiments will be necessary to understand more precisely the effect of other Smads and/or co-Smads proteins during TGF-β-induced apoptosis. Other investigations will also be required to better understand the specific roles of TGF-β2 and TGF-β3 at the intracellular and molecular level *in vitro *to determine how and if these isoforms control cell survival through Smad signal transducers.

## Authors' contributions

CS drafted the paper. CS, PLC, GFF, VL and MCD performed the experiments. EA conceived the study, participated in its design and coordination, and wrote final version of the manuscript. All authors read and approved the final manuscript.
